# Young and computer-literate healthcare professionals have the greatest expectations for heart failure telemonitoring

**DOI:** 10.1093/ehjdh/ztaa001

**Published:** 2020-11-30

**Authors:** Edita Lycholip, Ina Thon Aamodt, Irene Lie, Ragnhild Hellesø, Toma Šimbelytė, Roma Puronaitė, Anna Stromberg, Tiny Jaarsma, Jelena Čelutkienė

**Affiliations:** 1 Institute of Clinical Medicine, Faculty of Medicine, Vilnius University, Santariskiu str. 2, LT08661, Vilnius, Lithuania; 2 Department of Cardiothoracic Surgery, Centre for Patient-Centered Heart and Lung Research, Oslo University Hospital, Ullevål, Oslo, Norway; 3 Department of Nursing Science, Institute of Health and Society, University of Oslo, Oslo, Norway; 4 Lovisenberg Diaconal University College, Oslo, Norway; 5 Clinic of Internal Medicine, Centre of Family and Internal Medicine, Vilnius University Santaros Clinics, Vilnius University, Vilnius, Lithuania; 6 Cognitive Computing Group, Faculty of Mathematics and Informatics, Institute of Data Science and Digital Technologies, Vilnius University, Akademijos st. 4, 08412 Vilnius, Lithuania; 7 Division of Nursing, Department of Health, Medicine and Caring Sciences, Linköping University, Linköping, Sweden; 8 Department of Cardiology, Linköping University, Linköping, Sweden

##  

Telemonitoring (TM) is increasingly used in chronic disease management, for example, to monitor symptoms in patients with heart failure (HF) or atrial fibrillation, or to monitor battery status and alarms in cardiac implantable electronic devices. However, for successful implementation of remote TM in daily practice, the attitude of healthcare professionals (HCPs) to the benefits of using it is very important. There are only a few studies that explore experience and expectations of HCPs when using remote monitoring for the follow-up of HF patients.^1–3^ Despite clinical TM benefits, some HCPs who had experience with this technology, were disappointed with TM^1^ and mentioned an increase in workload.^2^^,^^3^ Healthcare providers have a direct role in applying TM and therefore it is important to identify the characteristics of HCPs associated with better expectations for TM.

We examined the characteristics of HCPs with the highest motivation for using TM. We pooled data from two studies^4^^,^^5^ in three Nordic-Baltic countries (Lithuania, Norway, and Sweden), which used cross-sectional surveys designed to investigate experiences and expectations of HCPs of remote non-invasive HF TM.^4^^,^^5^ In total, 647 nurses and cardiologists responded to this survey, 310 from Lithuania, 226 from Norway, and 120 from Sweden (*Table 1*).

**Table 1:** The number of responders in relation to the number of contacted HCPs

Country	Cardiologists Responder/contacted	Nurses Responder/contacted
Lithuania	137/201	173/254
Norway	63/109	163/220
Sweden	39/119	81/238

We defined the HCPs having the most positive expectations for TM in the future as those with the highest score in the question about 10 possible reasons for introducing TM; each reason was rated on a scale from 0 to 10, giving a total score from 0 to 100. Reasons for TM included: offering higher-quality care; reducing costs; implementing the vision/goal of the hospital; ability to treat more patients; reducing the workload on the HF out-patient clinic; reduce admissions/readmissions; better adherence to HF guidelines; improve self-care of HF patients; our centre is innovative; it`s mainly our Health authorities thinks that it’s important.

A regression tree analysis (*Figure 1*) revealed that younger HCPs (under 39 years of age), and among them the Lithuanian cardiologists and nurses with a work experience of less than 9 years, shared the most positive expectations (Node 4 in *Figure 1*, score 81); younger Norwegian and Swedish specialists also showed considerable enthusiasm (Node 6 in *Figure 1*, score 65). Meanwhile, in the older group of HCPs Lithuanian cardiologists and nurses with longer software experience (such as Word, PowerPoint, and Excel) showed higher expectation level (Node 9 in *Figure 1*, score 67). Among the participants of this older group, the Norwegian and Swedish nurses with longer software experience were slightly more enthusiastic (by the higher sum of all selected answers) (Node 13 in *Figure 1*, score 60) than the physicians of these two countries (Node 14 in *Figure 1*, score 58).

**Figure 1:**
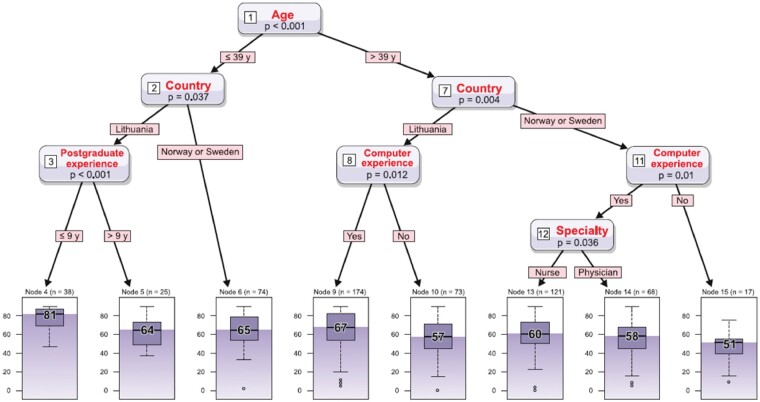
Regression tree showing HCPs characteristics with the greatest expectations for TM in HF patients. *Inputs*: Questions: Age; Country; What is your current job; How many years of postgraduate experience do you have; Sex; Are you familiar with heart failure telemonitoring; Do you have experience with programmes such as Word, PowerPoint, Excel. *Output*: The sum of all scores chosen in the question about importance of TM and expressed as median value with IQR (0–100). Central lines represent median scores, boxes represent the 25th and 75th percentiles, and whiskers extend to the lowest data point within 1.5 IQR of the lower quartile and the highest data point within 1.5 IQR of the upper quartile. Dots represent extreme values.

This data are important to consider when starting the introduction of TM in practice. The previously reported HCPs disappointment after daily experience with TM^1^ needs to be taken seriously and our data shows that characteristics of HCPs, such as age and experience in using digital technologies should be taken into account when considering implementation of TM in everyday HF practice.

In addition, only good expectations are not a sufficient factor to improve the adoption of TM. It is also essential to conduct appropriate training of HCPs on the use of the TM system, providing the technical assistance and the necessary infrastructure.^6^^,^^7^

The connection of these elements holds promise for swift implementation of TM in daily clinical practice.

## Data availability

Data are available upon request.

**Conflict of interest:** none declared.
